# Eczema in Babies

**Published:** 1909-08-07

**Authors:** 


### Dermatology.

### ECZEMA IN BABIES.

Infantile Eczema is one of the most troublesome complaints with which a physician has to deal, the reason being that no eczema will get well unless protected from local irritation, and in babies it is a very difficult matter to prevent rubbing and scratching. Eczema is a common complaint in infants of from three months to two years of age, and in these patients it always presents a very characteristic distribution. It is said to occur upon the buttocks in babies, but none of the various symptoms to which this region is liable in infants is really eczema. True eczema—an eruption marked by a red, more or less swollen condition of the skin attacked, with closely set pin-point vesicles, excoriations, or crusts over this area, accompanied by intense itching, and showing a great tendency to exacerbation as the result of any local irritation—is seen, in infants, always upon the face. It may also attack other parts, especially the scalp, the forearms, and the legs below the thighs, and in rare instances it may even become almost generalised; but in every case it is present upon the face.

The distribution of this affection is remarkable. It generally begins upon the cheeks and forehead as a diffuse redness and roughness of these parts, with minute vesication, or with crackling and oozing. It then spreads more widely over the face and scalp, but, curiously enough, it never attacks the skin around the eyes, of the nose, or around the mouth, so that the general appearance is that of a mask, with holes for the nose and eyes and mouth. When the inflammation becomes more marked, as it very soon does, owing to the fact that the intense itching induces the infant to scratch the parts or to rub them violently against any object within reach, then there may be much crusting from the coagulation of the abundant serous discharge. Soon there appear patches of eczema upon the forearms, where the child rubs them against its face, and upon the calves, from the rubbing of one leg with the foot of the other. The cause of all this trouble is generally very difficult or impossible to arrive at. Often the babies are otherwise apparently in good health, and frequently no error in diet can be discovered; while, of course, very many babies are badly fed, insufficiently fed, or overfed, without getting eczema. It is not very unusual to find that before the attack the mother has been in the habit of using some sort of strong soap for washing the infant, and although this cannot be regarded as the cause of all eczema in infants, it is no doubt a very frequent exciting cause. But although we are so seldom able to find any internal cause, we know well that all local irritation keeps up the eczematous condition and increases it.

It is obvious, therefore, that in the treatment of a case of infantile eczema, the main thing is to protect it from local irritation. If this can be secured absolutely for a few weeks the eczema will disappear, although no attempt has been made to deal with it by dieting or by internal remedies. The first thing is to avoid the intermittent application of water for washing purposes, and to keep the affected parts constantly protected by some simple dressing. If there is much crusting a continuous application of normal saline solution (sodium chloride ʒj. to Oj. of boiled water) on lint will be found useful until the crusts are removed, but in most cases it is sufficient to begin with a dressing of Lassar's paste (zinci oxidi ʒij., pulv. amyli ʒij., vaseline ℥ss.) spread upon a mask of fine butter-muslin and carefully bandaged on. This must be changed once or twice in twenty-four hours, removing the old dressing each time with liquid vaseline, and not with water. The greatest difficulty will be experienced in preventing the child from scratching or from rubbing its face against its arms or the bed-clothes, and means must be devised for fixing the head as much as possible and for tying up the hands. Sometimes, if the healing of an eczematous part is slow, it may be hastened by painting it with a 1 per cent. solution of nitrate of silver from time to time. There are probably many other simple protective applications which would answer as well as Lassar paste, the main object being to protect the affected areas from the irritation of scratching and rubbing.

